# Posterior wall ablation for persistent atrial fibrillation: Very-high-power short-duration versus standard-power radiofrequency ablation

**DOI:** 10.1016/j.hroo.2024.04.011

**Published:** 2024-04-26

**Authors:** Paolo Compagnucci, Giovanni Volpato, Laura Cipolletta, Quintino Parisi, Yari Valeri, Francesca Campanelli, Leonardo D’Angelo, Giuseppe Ciliberti, Giulia Stronati, Laura Carboni, Andrea Giovagnoni, Federico Guerra, Andrea Natale, Michela Casella, Antonio Dello Russo

**Affiliations:** ∗ Cardiology and Arrhythmology Clinic, Marche University Hospital, Ancona, Italy; † Department of Biomedical Sciences and Public Health, Marche Polytechnic University, Ancona, Italy; ‡ Cardiac Surgery Anesthesia and Critical Care Unit, Marche University Hospital, Ancona, Italy; § Department of Radiology, University Hospital “Ospedali Riuniti,” Ancona, Italy; ‖ Department of Clinical, Special and Dental Sciences, Marche Polytechnic University, Ancona, Italy; ¶ Texas Cardiac Arrhythmia Institute, St. David's Medical Center, Austin, Texas; # Department of Interventional Electrophysiology, Scripps Clinic, San Diego, California; ∗∗ Department of Internal Medicine, Metro Health Medical Center, Case Western Reserve University School of Medicine, Cleveland, Ohio; †† Department of Biomedicine and Prevention, University of Rome Tor Vergata, Rome, Italy

**Keywords:** Left atrial posterior wall ablation, Microbipolar mapping, PentaRay, Persistent atrial fibrillation, QDOT Micro^TM^, Very-high-power short-duration ablation

## Abstract

**Background:**

Posterior wall ablation (PWA) is commonly added to pulmonary vein isolation (PVI) during catheter ablation (CA) of persistent atrial fibrillation (AF).

**Objective:**

The purpose of this study was to compare PVI plus PWA using very-high-power short-duration (vHPSD) vs standard-power (SP) ablation index-guided CA among consecutive patients with persistent AF and to determine the voltage correlation between microbipolar and bipolar mapping in AF.

**Methods:**

We compared 40 patients undergoing PVI plus PWA using vHPSD to 40 controls receiving PVI plus PWA using SP. The primary efficacy endpoint was recurrence of atrial tachyarrhythmias after a 3-month blanking period. The primary safety outcome was a composite of major complications within 30 days after CA. In the vHPSD group, high-density mapping of the posterior wall was performed using both a multipolar catheter and microelectrodes on the tip of the ablation catheter.

**Results:**

PVI was more commonly obtained with vHPSD compared to SP ablation (98%vs 75%; *P* = .007), despite shorter procedural and fluoroscopy times (*P* <.001). Survival free from recurrent atrial tachyarrhythmias at 18 months was 68% and 47% in the vHPSD and SP groups, respectively (log-rank *P* = .071), without major adverse events. The vHPSD approach was significantly associated with reduced risk of recurrent AF at multivariable analysis (hazard ratio 0.39; *P* = .030). Microbipolar voltage cutoffs of 0.71 and 1.69 mV predicted minimum bipolar values of 0.16 and 0.31 mV in AF, respectively, with accuracies of 0.67 and 0.88.

**Conclusion:**

vHPSD PWA plus PVI may be faster and as safe as SP CA among patients with persistent AF, with a trend for superior efficacy. Adapted voltage cutoffs should be used for identifying atrial low-voltage areas with microbipolar mapping.

## Graphical abstract

**Figure:**
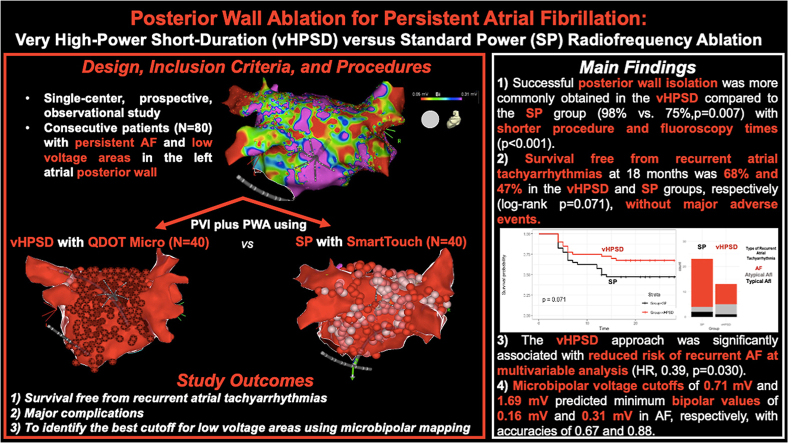

**Key Findings**

▪ Eighty patients with persistent atrial fibrillation (AF) and left atrial posterior wall (PW) low-voltage areas underwent pulmonary vein isolation plus PW ablation using either very-high-power short-duration (vHPSD) (n = 40) or standard power (SP) radiofrequency ablation (n = 40).
▪ Compared to SP, vHPSD was associated with a higher rate of PW isolation (98% vs 75%; *P* = .007) and shorter procedural/fluoroscopy times. No major adverse events were observed, including no cases of esophageal injury.
▪ At 18 months, survival free from atrial tachyarrhythmia recurrence occurred in a nonsignificantly lower number of patients in the vHPSD group than the SP group (68% vs 47%, respectively; *P* = .071). Considering the individual components of the primary endpoint, recurrent AF occurred less commonly with vHPSD.
▪ Microelectrode mapping is far more sensitive than standard bipolar mapping in AF. Microbipolar voltage cutoffs of 0.71 and 1.69 mV (previously validated cutoffs for atrial low voltage in AF) were found to predict minimum bipolar values of 0.16 and 0.31 mV, with accuracies of 0.67 and 0.88, respectively.

## Introduction

Catheter ablation (CA) has become an increasingly widespread treatment option for patients with persistent atrial fibrillation (AF). Although pulmonary vein isolation (PVI) has been shown to reduce arrhythmic recurrences and improve quality of life in patients with paroxysmal AF,^1^ persistent AF recognizes different mechanisms, which are strongly linked to atrial cardiomyopathic changes, and PVI alone is associated with suboptimal outcomes in this patient population.^1^, ^2^, ^3^ Therefore, the ablation of additional atrial structures potentially implicated in persistent AF initiation or perpetuation, such as the posterior wall (PW) and the left atrial (LA) appendage, has been investigated as a tool to improve the efficacy of CA,^3^^,^^4^ at the expense of lesser degree of procedural standardization and higher concerns of procedural complications.

Recently, a novel catheter with 3 microelectrode and 6 thermocouples (QDOT Micro^TM^, Biosense Webster, Diamond Bar, CA), which allows high-resolution mapping and very-high-power short-duration (vHPSD), temperature-controlled CA was developed and clinically tested in the setting of PVI for paroxysmal AF^5^, ^6^, ^7^ as well as for ventricular mapping.^8^ The vHPSD ablation mode was found to allow very rapid PVI, while limiting procedural complications by producing wider but shallower ablation lesions.^5^, ^6^, ^7^

In this study, we sought to investigate whether use of the vHPSD ablation mode impacts the procedural, safety, and efficacy outcomes of patients with persistent AF undergoing PVI plus posterior wall ablation (PWA) compared to standard power (SP), contact force–sensing, and ablation index (AI)–guided radiofrequency (RF) CA.^9^, ^10^, ^11^ Furthermore, we aimed to formally assess the impact of microelectrode mapping on characterization of the atrial substrate by exploring the voltage correlation between microbipolar and standard bipolar mapping.^8^^,^^12^

## Methods

### Study population

This was a single-center, prospective study that included consecutive patients undergoing CA of persistent AF with PVI plus PWA at the Marche University Hospital, Ancona, Italy, between June 2019 and February 2022. The reported research was performed according to institutional standards, national legal requirements, and the Declaration of Helsinki, and the patient data were prospectively collected in an institutional review board–approved database. Each patient provided informed consent.

The study included consecutive patients at least 18 years of age with symptomatic persistent AF, who were candidates for AF CA according to current clinical practice guidelines.^1^ The choice to perform PWA in addition to PVI was driven by the presence of low-voltage areas in the PW of the LA in each case, which were defined as the regions showing peak-to-peak bipolar voltage <0.31 mV in AF, a cutoff previously proposed as the best predictor of sinus rhythm voltage <0.5 mV.^12^ Exclusion criteria included severe untreated valvular heart disease and contraindications to CA because of intracardiac thrombus (Supplemental Methods).^1^ Patients undergoing PVI only were not included. For each patient, we collected demographics, clinical information, procedural details, and follow-up data.

### Study groups and CA procedure

Enrolled patients were categorized into 2 groups according to the technology and procedural approach adopted: (1) vHPSD group, which included consecutive patients undergoing PVI plus PWA using vHPSD RF CA with QDOT Micro; and (2) SP group, which included consecutive patients undergoing PVI plus PWA using SP, AI–guided RF CA with the ThermoCool SmartTouch ablation catheter (Biosense Webster). QDOT Micro became available in September 2021, and the choice of ablation technology after that date was based on operator’s preference, with 40 patients undergoing vHPSD ablation, and 17 patients receiving SP. Ablation of other structures beyond the pulmonary veins (PVs) and PW, such as the coronary sinus and LA appendage, was allowed at the operator’s discretion in both groups. (Further details are given in the Supplemental Methods.)

**Figure 1:**
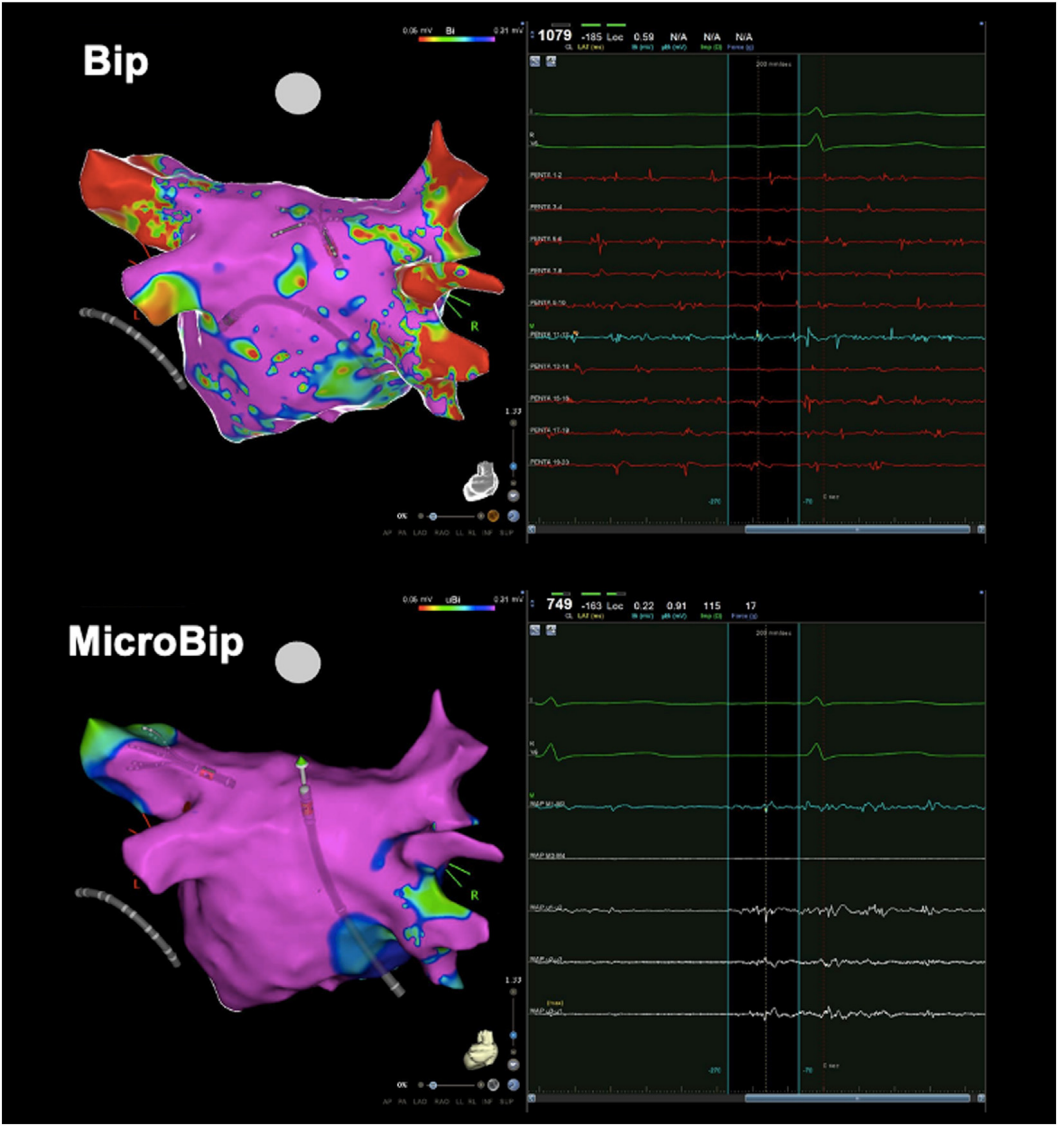
Comparison between minibipolar voltage mapping using the multipolar mapping catheter (PentaRay) (Bip), and microbipolar voltage mapping using microelectrodes on the ablation catheter’s (QDOT Micro) tip (MicroBip). Although low-voltage regions are recorded by bipolar mapping using a multipolar catheter, electrogram recorded by microelectrodes have normal amplitude in the same regions.

High-density electroanatomic reconstructions of the LA in AF were obtained by fast anatomic mapping using a multipolar catheter (PentaRay, Biosense Webster) and the ablation catheter. In the vHPSD group, special care was applied to obtain electroanatomic voltage maps of the PW by acquiring points with both the multipolar and the ablation catheters (Figure 1) in order to determine the best voltage cutoff with microelectrode mapping (using the highest voltage value detected with 1 of the 3 microelectrodes on the ablation catheter’s tip at each mapping location – microbipolar voltage) able to predict minibipolar voltages (as detected by the multipolar catheter) of 0.31 and 0.16 mV.^12^ These cutoffs were previously reported as an accurate cutoff for identifying atrial low-voltage regions in AF in the overall LA and in the LA PW, respectively.^12^ Only point pairs (ie, one acquired with the multipolar catheter and one with the ablation catheter) with a distance <1 mm between them were considered for analysis.^12^ For sensitivity analysis, the same process was performed for comparison of microbipolar and standard bipolar (ie, as recorded between the distal 3.5-mm tip and proximal 1-mm ring electrodes of the QDOT Micro catheter) voltages (see Supplemental Methods).

In both the vHPSD and the SP groups, antral PVI was performed using point-by-point RF applications around the PV ostia and in the intervenous carina.^9^ In the vHPSD group, the ablation settings were 50 W (QMODE, Biosense Webster) with an AI target of 550 for the anterior and superior segments of the PVs,^9^ whereas vHPSD RF applications (QMODE+; 90 W for 4 seconds, 8 mL/min flow rate; ablation stopped automatically if temperature increased to >65ºC cutoff) were delivered in posterior and inferior segments of the PVs. The recommended interlesion distance was 6 mm.^6^

In the SP group, antral PVI was performed using the ThermoCool SmartTouch ablation catheter.^6^ The ablation settings were 35 W with an AI target of 500–550 for the anterior and superior segments of the PVs, and 400 for the posterior and inferior segments of the PVs. Interlesion distance was set at 6 mm.^6^

**Figure 2:**
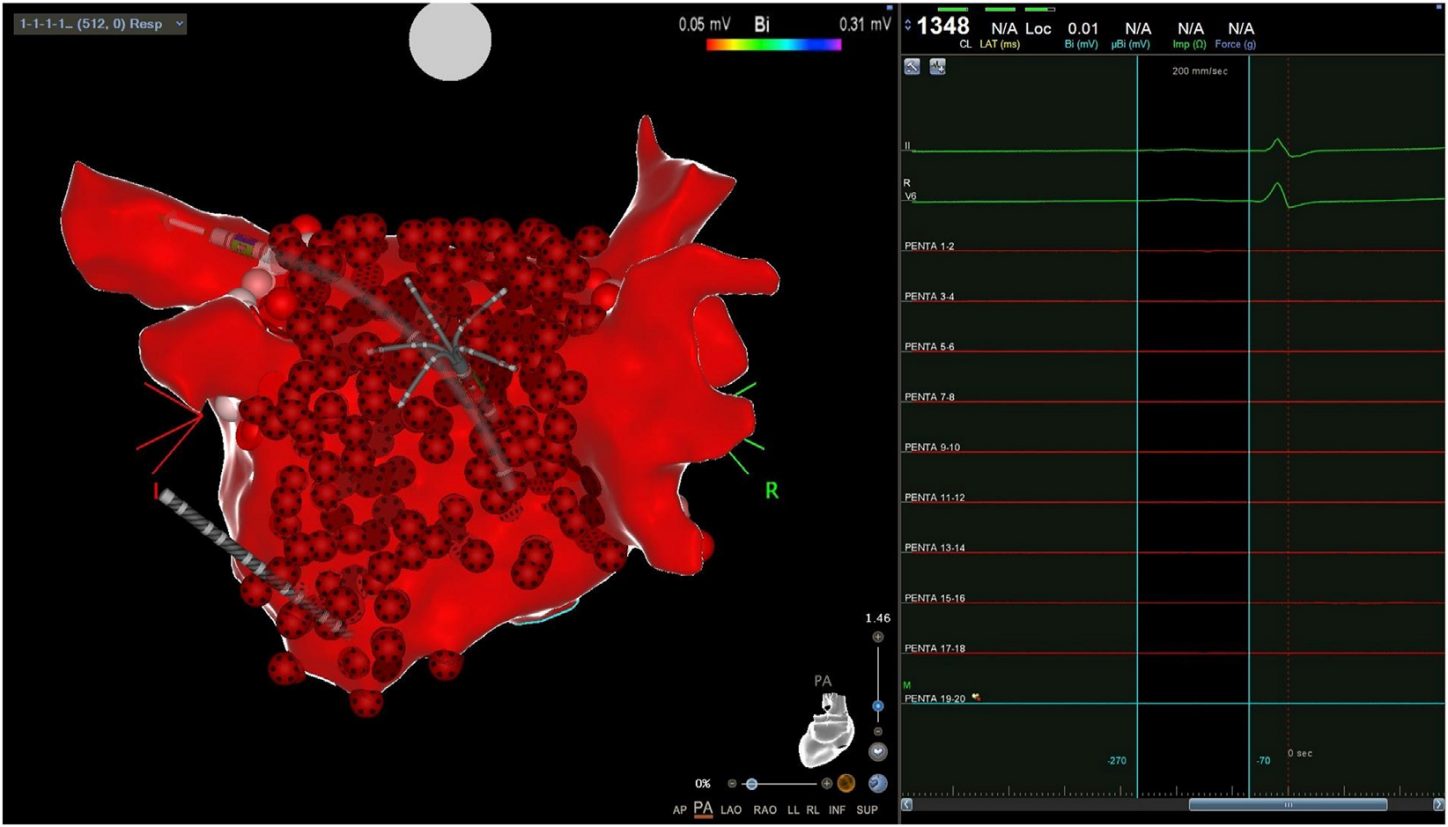
Very-high-power short-duration catheter ablation of the left atrial posterior wall. Note the multiple ablation tags covering the posterior wall, leading to posterior wall isolation as verified by remapping with a multipolar catheter.

After completion of PVI, PWA was performed in both groups by covering the PW with ablation lesions, targeting each zone of the PW displaying electrical activity (Figure 2). In the vHPSD group, RF applications were delivered with vHPSD ablation (QMODE+). In the SP group, the ablation settings for PWA were 35–40 W for 10 seconds. When concurrent coronary sinus ablation was performed, the most inferior portion of the PW close and along the course of the coronary sinus was ablated. Throughout the ablation procedure, esophageal temperature was monitored using an esophageal temperature probe, and ablation was interrupted as soon as the esophageal temperature reached 39°C. Additional RF applications were not delivered on posterior LA regions until the esophageal temperature reached baseline level.

Twenty minutes after completion of ablation, persistent PVI and PW isolation were confirmed by remapping in each PV and in the PW. Furthermore, exit block was confirmed in each PV by pacing with 10 mA at 2-ms pulses. For PV and PW touchups, operators used the vHPSD or the SP ablation mode, according to the study group.

### Patient follow-up and clinical outcomes

When available, implantable loop recorder/cardiac implantable electronic devices and remote monitoring were used to assess arrhythmia recurrence. Furthermore, 24-hour Holter monitoring (except in patients with implantable loop recorder/cardiac implantable electronic device), 12-lead electrocardiography, and clinical appointments were scheduled at 3 months after CA and every 6 months thereafter.^13^ Discontinuation of any antiarrhythmic drugs was recommended 3 months after CA in the absence of recurrent atrial tachyarrhythmias during the blanking period.

The primary efficacy outcome of the study was freedom from any atrial tachyarrhythmia (AF, atrial flutter, and atrial tachycardia) recurrences ≥30 seconds during follow-up after a 3-month blanking period. The primary safety outcome included the occurrence of any major complications (pericardial effusion/tamponade, stroke/transient ischemic attack, esophageal complications [especially atrioesophageal fistula], vascular complications, bronchial/respiratory complications, PV stenosis) during hospitalization and within the first 30 days of follow-up.

Survival free from AF recurrence and atrial tachyarrhythmia recurrence off antiarrhythmic drugs were key secondary efficacy outcomes. Acute procedural success was defined as successful PVI and PW isolation at the end of the procedure, as confirmed by documented persistent entrance block. Procedural outcomes included total procedural time, fluoroscopy time, and ablation time required to perform PWA.

### Statistical analysis

Continuous variables were checked for normality using the Shapiro-Wilk test and are given as mean ± SD if normally distributed, or as median [interquartile range] if non-normally distributed. Categorical variables are given as count (%).

To evaluate the best cutoff using microbipolar mapping for identifying atrial low-voltage regions as detected by minibipolar mapping (ie, those regions with minibipolar voltage <0.31/<0.16 mV in AF),^12^ we adjusted a generalized additive mixed model (GAMM), considering the patient as an independent random effect and using flexible penalized splines to model the continuous covariate. We calculated the threshold in microbipolar voltage corresponding to minimum minibipolar voltages of 0.31 and 0.16 mV when using the lower band of the 95% confidence interval for a given predicted mean minibipolar voltage.^12^ Then, to evaluate the performance of these thresholds, minibipolar voltages were predicted using the GAMM and compared with the actual bipolar voltages. Using the proportion of predicted bipolar voltages falling into the same categories (either <0.31/≥0.31 mV or <0.16/≥0.16 mV) as their measured bipolar corresponding, sensitivity, specificity, false positive/negative ratios, and accuracy were calculated. The same analysis was performed to compare microbipolar and standard bipolar mapping (see Supplemental Methods).

Comparisons of clinical/procedural characteristics between study groups were performed using χ^2^ test, Student *t* test, or Mann-Whitney *U* test, as appropriate. The time to primary outcome events was assessed with the Kaplan-Meier method, and comparisons between study groups were computed with the log-rank test. For sensitivity analysis, Cox proportional hazard regression models were fitted to identify predictors of outcome events. If any variable was associated with primary outcome events at *P* <.10 in the univariable analysis, the variable was also considered for inclusion in a multivariable model. All analyses were performed using R (R Core Team, Vienna, Austria). *P* <.05 was considered significant.

## Results

### Patient characteristics

During the study period, 40 consecutive patients underwent vHPSD CA with PVI and PWA, and 40 consecutive patients underwent SP CA. Clinical characteristics of patients at baseline were well balanced between the study groups (Table 1).

**Table 1:** Clinical and echocardiographic characteristics of the patients at baseline

	vHPSD group	SP group	*P* value
No. of patients	40 (100)	40 (100)	1
Age (y)	63 ± 9	62 ± 9	.540
Male	35 (88)	33 (83)	.754
Persistent AF episode duration (mo)			
<3	11 (28)	19 (48)	.106
3–12	23 (58)	19 (48)	.502
>12	6 (15)	2 (5)	.263
BMI (kg/m^2^)	27.3 ± 4.2	28.8 ± 3.8	.102
Indexed LA volume (mL/m^2^)	43 [36–51]	42 [36–49]	.935
LV ejection fraction (%)	55 [50–58]	51 [42–60]	.269
LV end-diastolic volume (mL/m^2^)	52 [47–59]	56 [46–63]	.482
Mitral regurgitation grading			
Absent/trivial	5 (13)	6 (15)	1
Mild	22 (55)	23 (58)	1
Moderate	13 (33)	11 (28)	.807
Congestive heart failure	5 (13)	6 (15)	1
Coronary artery disease	2 (5)	5 (13)	.432
Arterial hypertension	28 (70)	29 (73)	1
Diabetes mellitus type 2	4 (10)	3 (8)	1
CHA_2_DS_2_-VASc score			
0	6 (15)	3 (8)	.481
1	8 (20)	12 (30)	.439
2	9 (23)	7 (18)	.780
3	12 (30)	11 (28)	1
≥4	5 (13)	7 (18)	.756
First procedure	32 (80)	29 (73)	.599
CIEDs			
Overall	8 (13)	15 (38)	.138
Implantable loop recorder	6 (8)	11 (28)	.274
Dual-chamber pacemaker	1 (3)	0 (0)	1
ICD	1 (3)	4 (10)	.359

Values are given as n (%), mean ± SD, or median [1st–3rd quartile] unless otherwise indicated.

AF = atrial fibrillation; BMI = body mass index; CIED = cardiac implantable electronic device; ICD = implantable cardioverter-defibrillator; LA = left atrium; LV = left ventricle; SP = standard power; vHPSD = very-high-power short-duration.

### Procedural data

Procedural details are given in Table 2. All procedures were performed by operators with >10 years of experience in CA of AF. PVI was successfully obtained in all patients, with very high first-pass isolation rates in both groups (vHPSD 96%; SP 97%; *P* = 1) but with much shorter RF times in the vHPSD group (*P* <.001 for each PV). PW isolation was successfully confirmed by remapping at procedure end in a higher proportion of patients in the vHPSD group than in control patients (vHPSD 98%; SP 75%; *P* = .007). Furthermore, the ablated area in the PW was larger in the vHPSD group (16.9 ± 1.4 cm^2^) than the SP group (13.8 ± 1.1 cm^2^) (*P* < .001), despite a significantly shorter RF time (vHPSD 151 [120–188] seconds; SP 370 [338–428] seconds; *P* <.001). Of note, an average AI of 305 (280–350) was reached in the PW of patients in the SP group. Significantly shorter total procedural (vHPSD 81 [74–88] minutes; SP 198 [158–210] minutes; *P* <.001) and fluoroscopy times (vHPSD 09:30 [9–12:30] minutes; SP 15:30 [12–22] minutes; *P* <.001) were observed in the vHPSD group compared to control patients.

**Table 2:** Procedural details

	vHPSD group	SP group	*P* value
Skin-to-skin procedural time (min)	81 (74-88)	198 (158-210)	<.001
Total fluoroscopy time (min)	09:30 [9–12:30]	15:30 [12–22]	<.001
No. of PVs	160	160	
Total no. of isolated PVs	160 (100)	160 (100)	1
First-pass PVI			
Overall	154 (96)	155 (97)	1
LSPV	37 (93)	37 (93)	1
LIPV	40 (100)	40 (100)	1
RSPV	38 (95)	39 (98)	1
RIPV	39 (98)	39 (98)	1
Successful PW isolation at end procedure	39 (98)	30 (75)	.007
Ablation area in the PW (cm^2^)	16.9 ± 1.4	13.8 ± 1.1	<.001
RF time (s)			
LSPV	130 [108–241]	370 [340–394]	<.001
LIPV	70 [56–80]	300 [260–326]	<.001
RSPV	125 [112–134]	322 [310–345]	<.001
RIPV	70 [60–95]	270 [230–282]	<.001
Overall PVs	393 [345–450]	1220 [1173–1280]	<.001
PW	151 [120–188]	370[338–428]	<.001
No. of VisiTags			
Overall	123 ± 22	130 ± 17	.098
PVs	68 ± 64	74 [68–83]	.002
PW	38 [30–47]	35 [33–41]	.603
Overall average contact force *(g)*	10 [9–11]	10 [9–11]	.829
Ablation index			
PVs anterior/roof segments	550 [540–550]	520 [500–530]	<.001
PVs posterior/floor segments	—	400 [400–402]	
PW	—	305 [280–350]	
Other ablated structures			
Coronary sinus	13 (33)	8 (20)	.309
Left atrial appendage	5 (13)	7 (18)	.756
Cavotricuspid isthmus block	2 (5)	5 (13)	.432
Major complications	0 (0)	0 (0)	1
Minor complications	0 (0)	2 (5)	.494
Arteriovenous fistula	0 (0)	1 (3)	1
Transient postprocedural hypotension	0 (0)	1 (3)	1

Values are given as median [1st–3rd quartile], n (%), or mean ± SD or unless otherwise indicated.

LIPV = left inferior pulmonary vein; LSPV = left superior pulmonary vein; PV = pulmonary vein; PVI = pulmonary vein isolation; PW = pulmonary wall; RIPV = right inferior pulmonary vein; RSPV = right superior pulmonary vein.

### Comparison between microbipolar and bipolar mapping with potential microbipolar cutoff values for low-voltage areas

**Figure 3:**
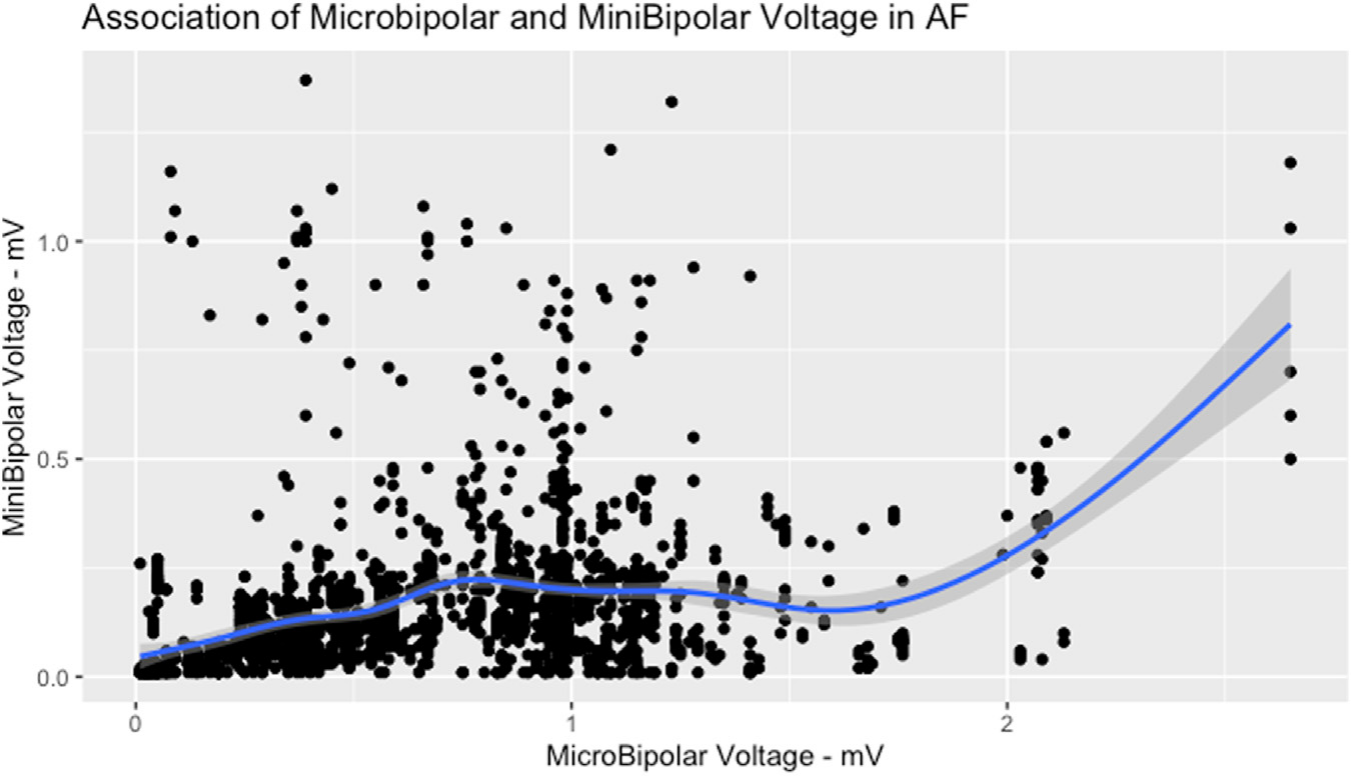
Comparison of the amplitude of maximum peak-to-peak microbipolar electrograms vs the amplitude of peak-to-peak minibipolar electrograms. AF = atrial fibrillation.

Among the 40 patients in the vHPSD group, a total of 2017 paired points were obtained from the LA PW in AF. The average voltage values with minibipolar and microbipolar mapping were 0.16 ± 0.18 mV and 0.69 ± 0.44 mV, respectively. The scatter plot of the association between microbipolar and minibipolar voltages is shown in Figure 3. An example of LA voltage maps using microbipolar and minibipolar mapping and the same voltage cutoffs is shown in Figure 1. The Kendall tau coefficient (Kendall rank correlation coefficient) was modest (tau = 0.309; *P* <.001) (Figure 3). By using GAMM, the best model yielded cutoffs of 0.71 and 1.69 mV using microbipolar mapping for predicting minimum values of 0.16 (0.16–0.51) mV and 0.31 (0.31–1.01) mV with minibipolar mapping, respectively. Sensitivity, specificity, positive/negative predictive values, and accuracy of these suggested cutoffs are given in Table 3. The comparison of microbipolar and standard bipolar mapping is given in the Supplemental Results, Supplemental Figure S1, and Supplemental Table S1.

**Table 3:** Assessment of performance of suggested microbipolar voltage cutoffs for predicting minibipolar low voltage

Definition of low minibipolar voltage	Microbipolar cutoff value	Min predicted minibipolar value	Mean predicted minibipolar value	Max predicted minibipolar value	SE	SP	PPV	NPV	Acc
<0.16 mV	0.71 mV	0.16 mV	0.17 mV	0.51 mV	0.66	0.69	0.78	0.56	0.67
<0.31 mV	1.69 mV	0.31 mV	0.34 mV	1.01 mV	0.99	0.12	0.89	0.53	0.88

Acc = accuracy; NPV = negative predictive value; PPV = positive predictive value; SE = sensitivity; SP = specificity.

### Safety and follow-up

No major adverse events occurred during the procedure and until the end of the first month of follow-up. No atrioesophageal fistulas or pericardial effusions/tamponade was observed. Only 2 patients, both in the SP group, developed 2 minor complications, which were managed conservatively (1 transient postprocedural hypotension and 1 small femoral arteriovenous fistula).

**Figure 4:**
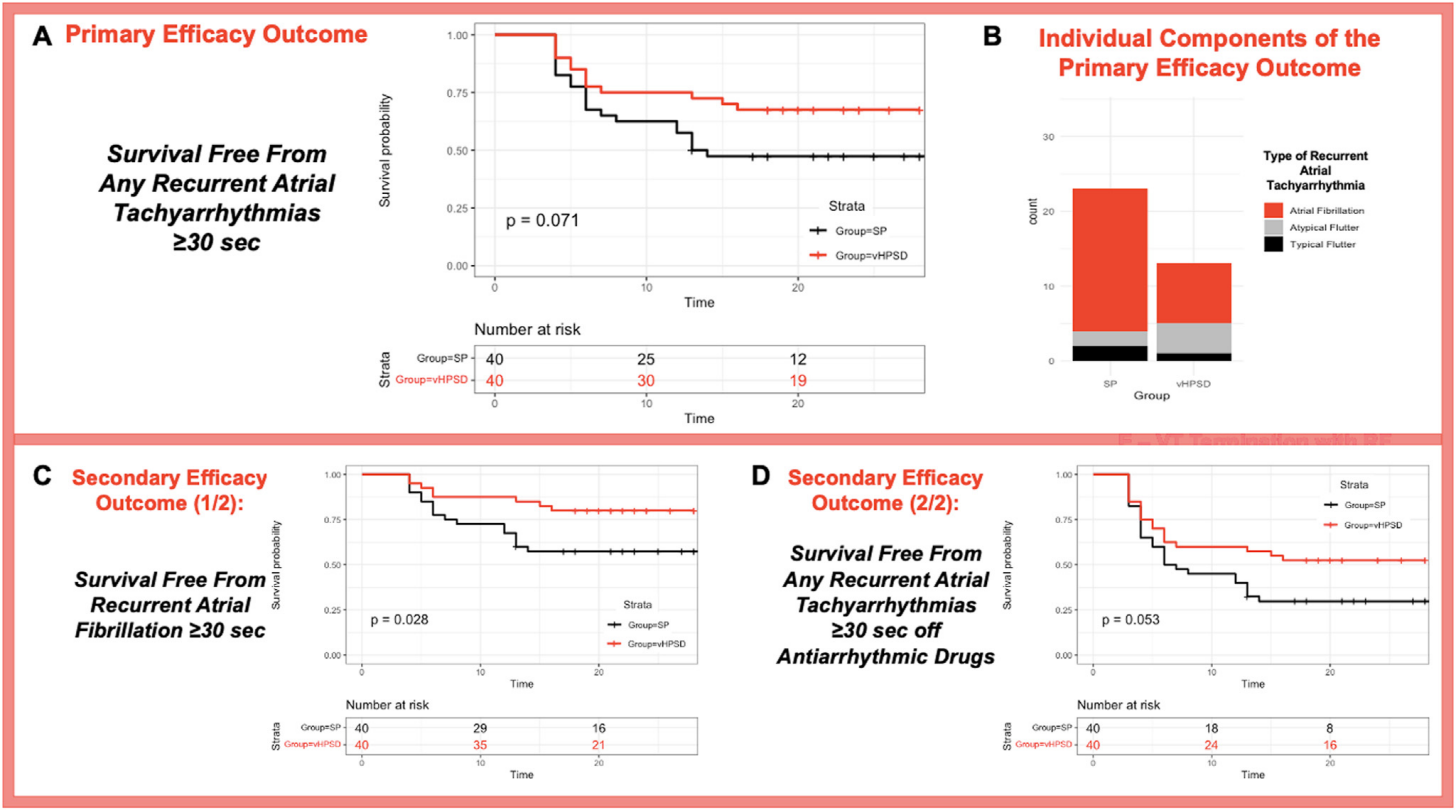
Survival free from overall recurrent atrial tachyarrhythmia during follow-up, individual components of the primary efficacy endpoint, and survival free from overall atrial tachyarrhythmia recurrence off antiarrhythmic drugs.

After median follow-up of 23 [20–26] months, there were nonsignificantly fewer atrial tachyarrhythmia recurrences in the vHPSD group (n = 12 [33%]) compared to the SP group (n = 23 [58%]; log-rank *P* = .071) (Figure 4A). Survival free from atrial tachyarrhythmia recurrence at 12 months was 0.75 (95% confidence interval [CI] 0.63–0.90) in the vHPSD group vs 0.58 (95% CI 0.44–0.75) in the SP group. At 18 months, survival free from atrial tachyarrhythmia recurrence was 0.68 (95% CI 0.54–0.84) in the vHPSD group vs 0.47 (95% CI 0.34–0.66) in the SP group. Considering the individual components of the primary endpoint (Figure 4B), AF recurrences were significantly more rare in the vHPSD group (n = 8 [20%]) compared to controls (n = 19 [48%) (log-rank *P* = .028) (Figure 4C), whereas risk of recurrent atypical and typical atrial flutter was similar between the groups (atypical flutter/atrial tachycardia—vHPSD: n = 4 [10%]; SP: n = 2 [5%]; log-rank *P* = .400; typical atrial flutter—vHPSD: n = 1 [3%]; SP: n = 2 [5%]; *P* = .550). A trend toward improved outcomes in the vHPSD group was obtained when analyzing survival free from recurrent atrial tachyarrhythmias off antiarrhythmic drugs (18-month Kaplan-Meier estimate of survival free from recurrent atrial tachyarrhythmias off antiarrhythmic drugs—vHPSD: 0.53 [95% CI 0.39–0.71]; SP: 0.30 [95% CI 0.18–0.48]; log-rank *P* = .053) (Figure 4D). A similar proportion of patients in both groups remained on antiarrhythmic drugs at 18-month follow-up (vHPSD: n = 19 [48%]; SP: n = 22 [55%]; *P* = .655).

At Cox multivariable regression analysis, based on alpha level of 0.10, a trend for an association of the ablation approach (vHPSD vs SP) with primary outcome events was observed, with vHPSD showing nonsignificantly lower risk (hazard ratio 0.54; 95% CI 0.27–1.09; *P* = .086) (Supplemental Table S2). Furthermore, the vHPSD ablation approach emerged as the only significant negative predictor of recurrent AF during follow-up (hazard ratio 0.39; 95% CI 0.17–0.91; *P* = .030) (Table 4).

**Table 4:** Univariable and multivariable Cox regression model for predicting risk of recurrent atrial fibrillation during follow-up

	Univariable analysis			Multivariable analysis		
	HR	95% CI	*P* value	HR	95% CI	*P* value
Age (per unit change)	1.00	0.96–1.04	.953			
Gender (male vs female)	0.70	0.26–1.87	.478			
Persistent AF episode duration (per month change)	1.06	0.94–1.19	.364			
BMI (per unit change)	1.00	0.91–1.10	.990			
Indexed LA volume (per unit change)	1.00	0.98–1.03	.936			
LV ejection fraction (per unit change)	0.99	0.96–1.02	.525			
LV end-diastolic volume (per unit change)	1.01	0.98–1.03	.673			
Mitral regurgitation grade						
Absent/trivial (yes vs no)	0.59	0.05–6.55	.668			
Mild (yes vs no)	0.48	0.05–4.36	.518			
Moderate (yes vs no)	0.83	0.10–7.22	.865			
Congestive heart failure (yes vs no)	1.78	0.67–4.75	.248			
Coronary artery disease (yes vs no)	2.51	0.86–7.32	.092	2.12	0.72–6.20	.172
Arterial hypertension (yes vs no)	1.11	0.47–2.64	.812			
Diabetes mellitus type 2 (yes vs no)	1.76	0.53–5.87	.361			
CHA_2_DS_2_-VASc score (per unit change)	1.07	0.79–1.43	.672			
Previous catheter ablation of AF (yes vs no)	0.74	0.28–1.96	.541			
CIED carrier status (carrier vs noncarrier)	2.21	0.99–4.87	.050	2.14	0.97–4.76	.061
Ablation approach (vHPSD vs SP)	0.40	0.17–0.93	.034	0.39	0.17–0.91	.030
Successful PW isolation at end procedure (yes vs no)	1.02	0.34–3.11	.966			
Ablation area in the PW (per unit change)	0.88	0.73–1.07	.209			
Ablation of other structures:						
Coronary sinus (yes vs no)	1.08	0.45–2.60	.855			
Left atrial appendage (yes vs no)	1.48	0.67–3.25	.335			
Cavotricuspid isthmus block (yes vs no)	1.00	0.30–3.40	.989			

CI = confidence interval; HR = hazard ratio; PW = posterior wall; other abbreviations as in Table 1.

## Discussion

### Main findings

To the best of our knowledge, this is the first report on the safety and efficacy of vHPSD PWA in addition to PVI among patients with persistent AF and evidence of low-voltage areas in the LA PW. Our findings suggest several main messages for clinicians:

1. Assessment of the PW substrate in AF is strongly dependent on the dimensions of the mapping electrodes. When using microbipolar mapping, higher cutoff values for the identification of low-voltage areas should be adopted, yet with suboptimal diagnostic performance for the prediction of low bipolar voltage according to published cutoffs.^12^
2. In our preliminary experience, vHPSD PWA proved to be fast, and we did not observe any cases of atrioesophageal fistula, pericardial effusion, or other major complications among patients undergoing vHPSD CA.
3. Compared to a control group of patients undergoing SP PWA plus PVI, patients undergoing vHPSD PWA plus PVI had nonsignificantly lower risk of atrial tachyarrhythmia recurrence during follow-up, both overall and off antiarrhythmic drugs, a result driven by a lower risk of recurrent AF.

### Microbipolar mapping of the LA

The recent introduction in clinical practice of ablation catheters equipped with submillimetric microelectrodes (0.167 mm^2^ for each of the 3 microelectrodes on the tip of the QDOT Micro catheter) has opened novel perspectives in electroanatomic mapping by allowing high-resolution mapping and ablation with a single device.^5^, ^6^, ^7^, ^8^ This novel possibility has been mainly explored in the ventricular milieu^8^^,^^14^^,^^15^ and for mapping the compact atrioventricular node,^16^ while a paucity of data is currently available on microbipolar atrial mapping. Our data suggest that microbipolar mapping is far more sensitive for the identification of viable atrial myocardium of the LA PW, and that higher voltage cutoffs need to be used for the identification of low-voltage regions, accordingly. Several factors may help to explain these findings. Microelectrodes have a smaller mapping surface, capturing the electrical activity of surviving myocardial bundles inside heterogeneous myocardial scars.^17^ Microbipolar electrograms are produced by the integration of electrical signals recorded between the 3 microelectrodes located on the catheter’s tip, which lie in direct contact with the endocardial surface, limiting the distance-related attenuation seen with mapping catheters equipped with electrodes lying along their linear splines, or with conventional bipolar mapping, in which electrograms are recorded between a linear catheter’s tip and a proximal ring electrode (usually not in contact with the endocardium).^14^ Furthermore, the electroanatomic mapping system automatically acquires the largest of the 3 microbipolar electrograms generated by each of the 3 microelectrodes, thus compensating for the direction of the propagating wavefront.^8^^,^^14^ Although we derived novel microbipolar voltage cutoffs for the identification of atrial low-voltage zones in AF, it is worth noting that there was significant variation in the association of bipolar and microbipolar voltage pairs at each mapping location, suggesting that, at present, bipolar voltage should be preferred for substrate assessment in AF and thus for guiding substrate-based AF ablation.

### vHPSD ablation beyond PVI

The recent introduction into clinical practice of ablation catheters allowing vHPSD ablation was built on important developments in the field of RF ablation for AF.^13^ Previous recognition of the importance of adequate catheter–tissue contact, as well as of catheter stability, led to the ideation, testing, and validation of contact force sensors,^18^ ablation lesions tagging systems, and RF lesion quality metrics,^9^ which increased the success rate and helped to standardize the procedural workflow of point-by-point RF PVI. vHPSD may represent an important additional step in achieving a balance between effectiveness and safety of PVI with RF energy. Use of higher power and irrigation settings may allow uniform and predictable ablation,^19^ via maximization of the diameter/depth ratio of individual ablation lesions, which may be especially desirable in thinner atrial regions and/or near the esophagus.^19^, ^20^, ^21^ The larger-diameter ablation lesions produced by vHPSD applications may also explain our finding of a larger PW ablation area in the vHPSD group despite a similar number of VisiTags^TM^ (Biosense Webster).^19^

Although vHPSD ablation was extensively tested in the setting of PVI,^5^, ^6^, ^7^ to the best of our knowledge this study represents the first systematic report on the use of this ablation technique outside PV antra and/or cavotricuspid isthmus among patients with AF.^5^, ^6^, ^7^^,^^22^^,^^23^ In our experience, no steam pops, atrioesophageal fistulas, pericardial effusions, or other major complications were observed despite performing PWA by covering the PW with vHPSD ablation lesions, suggesting that vHPSD may have an acceptable safety profile even in close proximity to the esophagus and in thin/diseased atrial regions (all patients enrolled had low-voltage areas in the PW), while significantly shortening the time required to complete PWA compared to SP ablation. Furthermore, a number of esophagus management strategies, including deviation and cooling, were recently found to be associated with reduced risk of esophageal damage after AF ablation using RF and may be applied to further increase procedural safety in future studies of PWA using vHPSD.^24^^,^^25^

### Impact of ablation settings on outcomes of PWA

The LA PW currently is regarded an important ablation target in efforts to improve CA success rate among patients with persistent AF.^2^^,^^3^ Several theoretical mechanisms have been proposed to explain why the addition of PWA to PVI theoretically may reduce the risk of recurrences after CA of persistent AF, including a common embryologic origin with PVs, electrophysiological/autonomic phenomena, the role of the PW as part of the critical mass necessary for maintaining AF, and the presence of heterogeneous scar in the PW.^26^ However, available evidence has been reported with heterogeneous and conflicting data on the efficacy of the addition of PWA to PVI among patients with persistent AF.^27^ In addition, the recent randomized controlled trial CAPLA (Catheter Ablation for Persistent Atrial Fibrillation: A Multicenter Randomized Trial of Pulmonary Vein Isolation vs PVI With Posterior Left Atrial Wall Isolation), in which PWA was mainly performed with SP ablation and by the deployment of floor and roof lines, respectively, connecting the inferior and superior PVs, failed to demonstrate any additional benefit of PVI plus PWA compared with PVI alone.^28^

The present study differs in several ways from CAPLA. In our study, the presence of low bipolar voltage regions in the PW (identified by high-density mapping and validated voltage cutoffs for AF^12^) was a prerequisite for study inclusion, whereas patients in CAPLA were randomized to PVI or PVI plus PWA irrespective of LA voltage maps.^28^ Accumulating evidences are reporting an association between atrial substrate modification by means of ablation of low voltage zones and improved outcomes in patients with persistent AF.^29^ Furthermore, CAPLA Investigators deployed linear RF ablation lesions (LA roof and floor lines), which may be prone to the development of gaps and/or recovery of conduction during follow-up.^28^ Indeed, the achievement of conduction block across ablation lines in the PW may be technically difficult and require multiple stacked or close RF applications, which might offset the advantage of a smaller ablation area associated with linear lesions in terms of esophageal safety.^30^ Finally, covering the PW with vHPSD ablation pulses may allow more homogeneous ablation of the PW, for which novel energy settings (such as vHPSD ablation) and/or sources (ie, electroporation) might be linked to improved outcomes.^31^^,^^32^

### Study limitations

First, this was a small, nonrandomized study with inherent risk of selection bias. The study was underpowered to detect rare complications (eg, atrioesophageal fistula, cardiac tamponade), and larger studies are necessary to confirm the safety of vHPSD PWA. Atrial substrate mapping was performed in AF, and the derivation of voltage cutoffs for low-voltage atrial myocardium using microelectrode mapping in sinus rhythm will require further work. The patients included in the study all had evidence of low-voltage myocardium in the LA PW, thus limiting the generalizability of our findings to other cohorts with preserved PW voltages. Although the proportion of patients with an implantable loop recorder was similar in the study groups, the majority of patients underwent intermittent monitoring during follow-up, leading to a potential underestimation of AF recurrences. Our study lacked a control group of patients undergoing PVI only, so conclusions can not be made on the additive value of PWA is persistent AF. Finally, in the SP group, low AI values were obtained in the PW to maximize safety; therefore, it is not possible to exclude that with higher AI targets, survival free from recurrent atrial tachyarrhythmias would have been more favorable in the SP group.

## Conclusion

Microbipolar mapping allowed the recording of higher voltage electrograms compared to bipolar mapping, thus underscoring the need to adapt the voltage cutoffs for identification of low-voltage atrial myocardium to the dimensions of mapping electrodes. We demonstrated for the first time that vHPSD CA might allow fast and acceptably safe PWA, with at least noninferior survival free from recurrent atrial tachyarrhythmias compared to SP PWA. Large, randomized studies are needed to confirm these promising findings.
