# Supplementary material for: Posterior wall ablation for persistent atrial fibrillation: Very-high-power short-duration versus standard-power radiofrequency ablation
Source: Heart Rhythm O2. 2024 Apr 26;5(6):374–84. doi: 10.1016/j.hroo.2024.04.011 (PMC11228279; doi:10.1016/j.hroo.2024.04.011)
Supplement: Supplementary Material [file mmc1.docx]

**SUPPLEMENTARY MATERIAL**

**Supplemental Methods** – page 2

**Supplemental Results** – page 3

**Supplemental References** – page 4

**Supplemental Figure S1** – page 5

**Supplemental Table S1** – page 6

**Supplemental Table S2** - page 7

**Supplemental Methods**

*Exclusion of intracardiac thrombus and management of oral anticoagulant therapy*

Prior to the procedure, intracardiac thrombus was always excluded with transesophageal echocardiography or cardiac computed tomography, in accordance with current clinical practice guidelines.^1,2^ In patients anticoagulated with vitamin K antagonists, the procedure was performed under therapeutic international normalized ratio values of 2–3. In patients on new oral anticoagulants, the morning dose on the day of the procedure was omitted, and anticoagulation was resumed in the evening, after the procedure.^2^

*Ablation of the cavotricuspid isthmus, coronary sinus, and left atrial appendage*

In both study groups, the cavotricuspid isthmus was ablated in all patients with history of typical flutter. In the vHPSD group, a cavotricuspid isthmus line was created by delivering radiofrequency pulses with 50Watt (QMODE) settings, while in the SP group ablation settings were 35-40 Watt, with applications prolonged until elimination of local electrograms. Cavotricuspid isthmus block was confirmed after ablation using standard pacing maneuvers.^3^

The additional ablation of coronary sinus and left atrial appendage (LAA) was based on operators’ preference. For coronary sinus, ablation settings were 20-35Watt for 10 seconds in both groups; ablation was started distally, and the ablation catheter was pulled back to the CS ostium, making sure that the catheter tip was freely moving, and not wedged in a small branch of coronary sinus.^4^ The procedural endpoint was the electrical isolation of the coronary sinus.^4^ For LAA ablation, settings were 50Watt (QMODE) in the vHPSD group, and 30-35Watt in the SP group; individual applications were continued until abolition of local electrograms, and the procedural endpoint was LAA electrical isolation.^5^ After LAA ablation, patients were permanently maintained on oral anticoagulation.^5^

**Supplemental Results**

*Comparison between MicroBipolar and Standard Bipolar Mapping With Potential MicroBipolar Cutoff Values For Low Voltage Areas*

Among the 40 patients in the vHPSD group, a total of 2209 points were mapped in the left atrial PW in AF using the QDOT Micro catheter. The average voltage values with standard bipolar and microbipolar mapping were 0.18 ± 0.37 mV and 0.73 ± 0.57 mV, respectively; the scatter plot of the association between microbipolar and standard bipolar voltages is represented in Supplementary Figure 1. The Kendall’s tau coefficient (Kendall rank correlation coefficient) was modest (tau=0.298; p<0.001), as seen graphically in the Supplementary Figure S1. By using GAMM, the best model yielded cutoffs of 1.01 mV and 2.09 mV using MicroBipolar mapping for predicting minimum values of 0.16 mV and 0.31 mV with standard bipolar mapping, respectively. The sensitivity, specificity, positive/negative predictive value, and accuracy of these suggested cutoffs are reported in Supplementary Table S1.

**Supplemental References**

1. Hindricks G, Potpara T, Dagres N, et al. 2020 ESC Guidelines for the diagnosis and management of atrial fibrillation developed in collaboration with the European Association for Cardio-Thoracic Surgery (EACTS): The Task Force for the diagnosis and management of atrial fibrillation of the European Society of Cardiology (ESC) Developed with the special contribution of the European Heart Rhythm Association (EHRA) of the ESC. Eur Heart J 2021;42:373-498.
2. Calkins H, Hindricks G, Cappato R, et al. 2017 HRS/EHRA/ECAS/APHRS/SOLAECE expert consensus statement on catheter and surgical ablation of atrial fibrillation. Heart Rhythm 2017;14:e275-e444.
3. Cauchemez B, Haissaguerre M, Fischer B, Thomas O, Clementy J, Coumel P. Electrophysiological effects of catheter ablation of inferior vena cava-tricuspid annulus isthmus in common atrial flutter. Circulation 1996;93(2):284-94.
4. Mohanty S, Trivedi C, Della Rocca DG, et al. Recovery of Conduction Following High-Power Short-Duration Ablation in Patients With Atrial Fibrillation: A Single-Center Experience. Circ Arrhythm Electrophysiol 2021;14(10):e010096.
5. Di Biase L, Burkhardt JD, Mohanty P, et al. Left Atrial Appendage Isolation in Patients With Longstanding Persistent AF Undergoing Catheter Ablation: BELIEF Trial. J Am Coll Cardiol 2016;68(18):1929-1940.

**
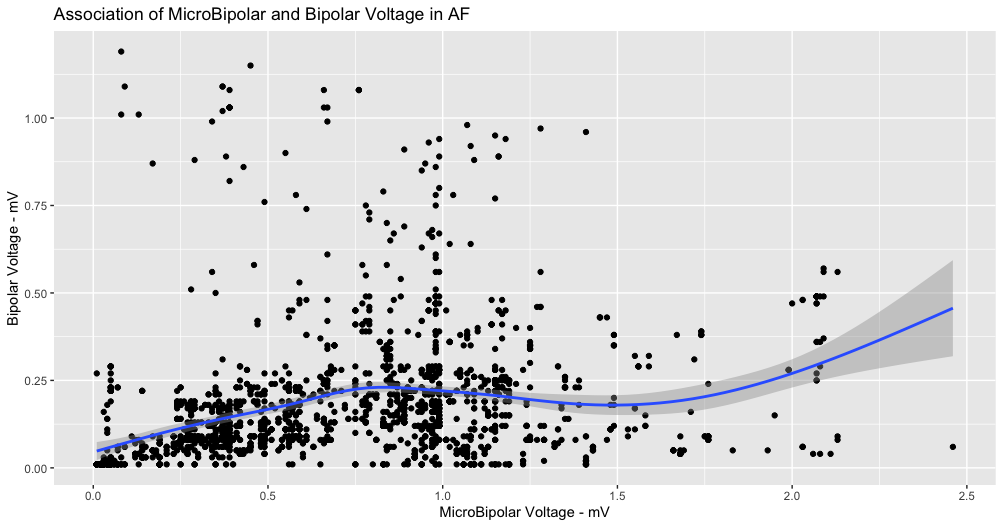
Supplemental Figure S1. Comparison of the amplitude of maximum peak-to-peak microbipolar electrograms versus the amplitude of peak-to-peak standard bipolar electrograms.**

**Supplemental Table S1. Assessment of the performance of the suggested Microbipolar voltage cutoffs for predicting standard bipolar low voltage**

| **Definition of Low Bip Voltage** | **MicroBip Cutoff Value** | **Min Predicted Bip Value** | **Mean Predicted Bip Value** | **Max Predicted Bip Value** | **SE** | **SP** | **PPV** | **NPV** | **Acc** |
| --- | --- | --- | --- | --- | --- | --- | --- | --- | --- |
| <0.16 mV | 1.01 mV | 0.16 mV | 0.18 mV | 0.19 mV | 0.51 | 0.81 | 0.78 | 0.56 | 0.64 |
| <0.31 mV | 2.09 mV | 0.31 mV | 0.35 mV | 0.40 mV | 0.99 | 0.05 | 0.88 | 0.70 | 0.88 |

**Supplemental Table S2. Univariable and multivariable Cox regression model for predicting risk of primary outcome events during follow-up.**

|  | **Univariable Analysis** | | | **Multivariable Analysis** | | |
| --- | --- | --- | --- | --- | --- | --- |
| **Variable** | **HR** | **95% CI** | **P value** | **HR** | **95% CI** | **P value** |
| Age (per unit change) | 0.99 | 0.95-1.02 | 0.492 |  |  |  |
| Gender (male vs female) | 0.84 | 0.35-2.03 | 0.698 |  |  |  |
| Persistent AF episode duration (per month change) | 0.99 | 0.89-1.11 | 0.894 |  |  |  |
| BMI (per unit change) | 1.04 | 0.96-1.13 | 0.388 |  |  |  |
| Indexed LA volume (per unit change) | 0.99 | 0.97-1.02 | 0.598 |  |  |  |
| LV ejection fraction (per unit change) | 1.00 | 0.97-1.03 | 0.978 |  |  |  |
| LV End Diastolic Volume (per unit change) | 0.99 | 0.97-1.02 | 0.577 |  |  |  |
| Mitral Regurgitation Grading:  Absent/Trivial (yes vs no)  Mild (yes vs no)  Moderate (yes vs no) | 1.69  0.79  0.82 | 0.17-16.42  0.09-7.00  0.09-7.15 | 0.650  0.836  0.856 |  |  |  |
| Congestive heart failure (yes vs no) | 1.09 | 0.42-2.82 | 0.858 |  |  |  |
| **Coronary artery disease (yes vs no)** | **4.02** | **1.65-9.77** | **0.002** | 2.29 | 0.77-6.81 | 0.137 |
| Arterial Hypertension (yes vs no) | 0.99 | 0.48-2.07 | 0.988 |  |  |  |
| **Diabetes mellitus type 2 (yes vs no)** | **3.13** | **1.21-8.13** | **0.019** | 2.59 | 0.80-8.41 | 0.113 |
| CHA_2_DS_2_-VASc score (per unit change) | 0.98 | 0.75-1.26 | 0.852 |  |  |  |
| Prior catheter ablation of AF (yes vs no) | 0.77 | 0.34-1.77 | 0.543 |  |  |  |
| **CIEDs carrier status (carrier vs non carrier)** | **1.82** | **0.90-3.67** | **0.093** | 1.84 | 0.90-3.77 | 0.097 |
| **Ablation approach**  **(vHPSD vs SP)** | **0.53** | **0.27-1.07** | **0.077** | 0.54 | 0.27-1.09 | 0.086 |
| Successful PWI at end procedure (yes vs no) | 1.07 | 0.40-2.83 | 0.899 |  |  |  |
| Ablation area in the PW (per unit change) | 0.90 | 0.76-1.06 | 0.208 |  |  |  |
| Ablation of other structures:  Coronary sinus (yes vs no)  Left atrial appendage (yes vs no)  Cavotricuspid isthmus block (yes vs no) | 1.18  1.68  0.97 | 0.56-2.46  0.85-3.30  0.34-2.78 | 0.665  0.136  0.959 |  |  |  |
